# The impact of surgeon’s experience and sex on the incidence of cystoid macular edema after uneventful cataract surgery

**DOI:** 10.1371/journal.pone.0279518

**Published:** 2022-12-27

**Authors:** Wolfgang List, Gernot Steinwender, Wilfried Glatz, Regina Riedl, Andreas Wedrich, Domagoj Ivastinovic

**Affiliations:** 1 Department of Ophthalmology, Medical University of Graz, Graz, Austria; 2 Institute for Medical Informatics, Statistics and Documentation, Medical University of Graz, Graz, Austria; University of Warmia, POLAND

## Abstract

**Purpose:**

To assess the rate of pseudophakic cystoid macular edema (pCME) in uneventful cataract surgery in surgeons in training vs experienced surgeons and to analyze the rate of pCME according to surgeon’s sex.

**Methods:**

Medical reports post phacoemulsification between 2010 and 2018 at the Department of Ophthalmology, Medical University of Graz, Austria, were reviewed for pCME. A running lifetime number of preceding cataract surgeries was used to express hands-on experience. A cut-off number of 300 surgeries was defined to distinguish between surgeons in training and experienced surgeons. Outcome parameters were incidence of pCME, patient’s sex and age, laterality of eye, coexistence of pseudoexfoliation syndrome (PEX), duration of surgery and surgeon’s sex.

**Results:**

25.422 surgeries on 18.266 patients were included. The majority was performed by experienced surgeons (23.139, 91.0%) vs 2.283 (9.0%) by surgeons in training (25 surgeons, 9 (36%) female and 16 (64%) male). pCME occurred in 32 eyes (1.4%) following surgery by surgeons in training and in 152 eyes (0.7%) following surgery by experienced surgeons. Chance for pCME was 1.57 higher in training surgeries (95% CI 1.03–2.41, p = 0.034) and longer duration (OR = 1.04; 95% CI 1.02–1.07, p = 0.001). After excluding the first 100 surgeries for every surgeon in training similar results were observed. No difference in risk for pCME was found between female and male surgeons in both groups (training and experienced surgeons).

**Conclusion:**

In conclusion, the rate for pCME after uneventful cataract surgery is significantly higher for surgeons in training but steadily decreasing and associated to surgical time. No difference in the risk for pCME was found between female and male surgeons.

## Introduction

Pseudophakic cystoid macular edema (pCME), also referred to as Irvine-Gass syndrome, was reported to occur in 0.2% to 2.35% after uncomplicated cataract surgery [1–4]. Due to the regular use of spectral domain optical coherence tomography (sdOCT) pCME appeared to be much more frequent, reaching up to 13.9% in recent years [2, 5–8]. The exact pathomechanisms of pCME are not yet fully understood, however, there are three main components assumed: breakdown of the blood-retina-barrier, intraocular inflammation with an increased release of proteins and prostaglandins into the intraocular milieu and mechanically induced forces on the vitreous. Appearance of these can be deduced to a more traumatic surgery and prolonged surgery duration–features that are likely to apply to surgeries performed by surgeons in training [9–18].

In previous years, a recommendation of 40 to 80 procedures was mentioned to achieve proficient surgery with low rates of complications [19–23]. In contrast, after analysis of the risk for posterior capsule rupture the German Ophthalmological Society (GOS) proposed that the first 300 cataract surgeries should be performed under supervision [24]. According to this proposal, we set the cut-off for surgeons in training versus experienced surgeons in our analysis to 300.

The association between complicated cataract surgery and appearance of pCME is already known [25–28]. Following posterior capsule rupture, the relative risk (RR) for pCME increases significantly by RR 2.61 to 5.05 [25, 29]. We therefore only included uneventful cataract surgeries to assess the rate of pCME in uneventful cataract surgery.

In the present study we assessed the rate of pCME in surgeons in training vs experienced surgeons in uneventful cataract surgery and analyzed the rate of pCME according to surgeon’s sex.

## Materials and methods

In this retrospective study, we reviewed all uneventful phacoemulsifications with intraocular lens (IOL) implantations at the Department of Ophthalmology, Medical University of Graz, between January 1^st^, 2010 and September 30^th^, 2018. During this study period, we carefully assessed all diagnosed clinically significant pCME in the postoperative course. A continuously running lifetime number of total preceding cataract surgeries for each surgeon was used to express their surgical hands-on experience. The counting began with the first cataract surgery performed, independent from the time period of this study. Surgeons who worked and operated on in other hospitals before were asked for the number of previously conducted cataract surgeries. Surgeries that were performed outside of our hospital remained unconsidered for this analysis. Altogether we accessed data from 25 cataract surgeons from our department during the study period.

Surgical and medical reports were thoroughly reviewed for presence of the following exclusion criteria: posterior capsule ruptures with and without vitreous body prolapses, zonular dialysis, iris trauma or intraoperative iris manipulation, intraocular hemorrhage during surgery, IOL implantation in aphakic eyes, combined procedures (IOL implantation with simultaneous glaucoma surgery or simultaneous vitrectomy), diabetes mellitus, prior retinal vein occlusions, history of uveitis and iritis, maculopathies and retinopathies of any kind and exudative macular degenerations, epiretinal membranes, presence of ocular tumors, implausible surgery documentation (surgery duration less than 3 minutes), as well as previous ophthalmic surgeries within the last three months. Data remained unconsidered if one or more was applicable. Only uneventful surgeries were considered. We therefore excluded all surgeries from analysis for the above-mentioned exclusion criteria, as well as surgeries with a duration of over 30 minutes (duration between the beginning of the surgery until the end of the surgery). Only patients with clinically significant CME were considered for this study if they were referred by registered ophthalmologists or diagnosed at the outpatient department.

This single-center study was conducted at the Department of Ophthalmology, Medical University of Graz. Ethics Committee approval was obtained by the Ethics Committee of the Medical University of Graz (approval number: 30–413 ex 17/18). The Ethics Committee of the Medical University of Graz ruled that written informed consent was not required for this study. Patient records were anonymised and de-identified prior to analysis. The study was conducted in accordance with the principles and regulations of the Declaration of Helsinki. Data was accessible for members of the project team solely.

### Study sample

A total of 33.319 cataract surgeries were performed during the study period. In consideration of inclusion and exclusion criteria, 25.422 surgeries from 18.266 patients were included in our analyses. The majority of surgeries was performed by experienced surgeons (23.139, 91.0%) while 2.283 (9.0%) surgeries were performed by surgeons in training. Data of 25 surgeons were included, 9 (36%) females and 16 (64%) males.

### Surgical training

Two groups were defined distinguishing between surgeons in training and experienced surgeons. The cut-off for surgeons in training was set for the first 300 surgeries. This cut-off was chosen according to the proposal of the German Ophthalmological Society (GOS), recommending the procedure of the first 300 cataract surgeries under supervision.

In addition, we also performed analysis excluding the first 100 surgeries as surgeons usually start off by performing only a few steps. Surgeons in training are generally assigned to and supervised by one or two experienced ophthalmic surgeons at all stages of training. The teaching strategy is determined by the experienced surgeon: first surgeries are either done step by step so that surgeons in training can start off by doing the incisions and capsulorhexis or by aspiration of the viscoelastics at the end of the surgery. Supervision is gradually eased by experienced surgeons as routine is gained.

### Surgical technique

All cataract surgeries were performed using small incision phacoemulsification technique and implantation of posterior chamber intraocular lenses. A local anesthesia was obtained by parabulbar injection of Oxybuprocain 0.4%, 2.5ml Ropivacain 10mg/ml + 2.5ml Lidocain 2%. Before corneal incision, povidone iodine 2% was instilled onto the cornea and conjunctiva. Intraoperative mydriasis was obtained by topical instillation of tropicamide 0.5% and phenylephrine 1.0% twice preoperatively and intracameral injection of adrenaline and lidocaine before capsulorhexis. The anterior chamber was stabilized by injection of methyl cellulose, capsulorhexis was performed with a needle.

Postoperative standardized therapeutic regimen in the study period consisted of glucocorticoid and antibiotic eye drops and ointment. A combination of betamethasone and neomycin sulfate eye drops (Betnesol N^®^, ALFASIGMA S.P.A., Italy; Betamethasone 1mg/ml, Neomycin sulfate 5mg/ml) was prescribed 5 times per day and tapered by one drop every week. For the first postoperative week, a combination of dexamethasone and gentamicin sulfate ointment at nighttime was additionally prescribed (Dexagenta^®^, Ursapharm GmbH, Austria; Dexamethasone 0.3mg/g, Gentamicin sulfate 5mg/g).

### Pseudophakic cystoid macular edema

Every pCME was related to a surgeon in training or an experienced surgeon, according to the surgeon’s running lifetime number of preceding cataract surgeries. In the course of the study, surgeons could advance from surgeons in training to experienced surgeons if they exceeded the 300 previous procedures.

pCME was defined as macular thickness over 300 μm and the presence of intraretinal hyporeflective cysts within the ETDRS (Early Treatment Diabetic Retinopathy Study) circle (central, concentric parafoveal area, 6.0 mm) [2, 8, 30]. Therefore, sdOCT (Spectralis version 6.0.9 software, Heidelberg Engineering, Heidelberg, Germany) was used with following adjustments: volumetric scanning with 25 sections covering a field of 20x20° in the macular region. A bandwidth of 297 nm and a wavelength of 815 nm was used [31, 32]. Eye movement was compensated using the eye tracking software (TruTrack) to remain exact positioning of the recorded scans [31]. The scanning was done using high-speed mode with a resolution of 7 μm axially and 14μm laterally, the in between distance for every section was set to 240 μm [32].

In addition to pCME, we additionally analyzed covariables including patient’s sex and age, side of the eye, coexistence of pseudo exfoliation syndrome (PEX), the duration of the surgery and surgeon’s sex.

### Statistical analysis

In descriptive statistics, continuous parameters are presented as median, minimum and maximum and categorical parameters as frequencies and percent. To evaluate the influence of surgeries with surgeons in training and experienced surgeons on occurrence of pCME, generalized estimating equations (GEE) with logit as link function and exchangeable working correlation structure accounting for repeated surgeries on the same patient was used [33]. The multivariable model includes pCME as dependent parameter and the surgeon’s training state (surgeon in training or experienced), patient’s sex (female/male), eye (left/right), coexistence of pseudo exfoliation syndrome (PEX: yes/no), patients age and duration (in minutes) of the procedure as independent parameters. The results are presented as odds ratios (ORs) with their corresponding 95% confidence interval (CI) and p-value. In sensitivity analyses, the model was repeated, but excluding the first 100 surgeries to rule out the influence of intervening supervision. In addition, a model, including the surgeon’s sex as an additional parameter was performed. For visualization purposes of a learning curve, a B-spline fit with 3 knots was performed by plotting pCME occurrence against the number of surgeon’s previous cataract operations. Statistical analyses were performed using SAS 9.4 (SAS Institute, Cary NC).

## Results

Characteristics of our study population in total and by training and experienced surgeries are summarized in Table 1.

**Table 1:** Characteristics of the study population in total and by surgeon in training and experienced surgeon.

Characteristic	Description	Total N = 25.422	Surgeon in training N = 2.283	Experienced Surgeon N = 23.139	p-value*
Age	(in years)	76.0 (50.0, 100.0)	77.0 (50.0, 97.0)	76.0 (50.0, 100.0)	< .001
Duration of Surgery (in min)	(in minutes)	11.1 (3.0, 30.0)	17.2 (5.9, 30.0)	10.8 (3.0, 30.0)	< .001
Patient’s sex	Female	15539 (61.1%)	1417 (62.1%)	14122 (61.0%)	0.337
	Male	9883 (38.9%)	866 (37.9%)	9017 (39.0%)	
Side	LE	12569 (49.5%)	1188 (52.0%)	11381 (49.2%)	0.005
	RE	12849 (50.6%)	1195 (48.0%)	11754 (50.8%)	
	Missing	4		4	
PEX	PEX	1953 (7.7%)	113 (4.9%)	1840 (8.0%)	< .001
	No PEX	23468 (92.3%)	2170 (95.1%)	21298 (92.0%)	
	Missing	1		1	
pCME	pCME	184 (0.7%)	32 (1.4%)	152 (0.7%)	-
	No pCME	25238 (99.3%)	2251 (98.6%)	22987 (99.3%)	

Abbreviations: LE: left eye, min: minute, N: absolute number, pCME: pseudophakic cystoid macular edema, PEX: pseudo exfoliation syndrome, RE: right eye.

*p-value from generalized estimating equations model

In training surgeries vs experienced surgeries, patients were older (median 77 (50–97) vs 76 (50–100) years), the duration of the surgery was longer (median 17.2 (5.9–30) vs 10.8 (3–30) minutes), patients were more commonly female (62.1% vs 61.0%), more left eyes were operated on (52.0% vs. 49.2%) and PEX was seen in 4.9% of eyes, compared to 8.0% in surgeries by experienced surgeons. In total, pCME was reported in 184 eyes (0.7%) after surgery, thereof in 32 eyes (1.4%) operated by surgeons in training and 152 eyes (0.7%) operated by experienced surgeons.

In multivariable analysis, 52 surgeries were excluded due to missing values. The results are presented in Table 2. We observed a higher risk for pCME for surgeons in training (OR = 1.57; 95% CI 1.03–2.40, p = 0.034) and longer duration (OR = 1.04; 95% CI 1.02–1.07, p = 0.001). Lower chances were observed for surgeries on left eyes (OR = 0.74; 95% CI 0.57–0.96, p = 0.022) and by trend for female patients (OR = 0.77; 95% CI 0.56–1.06, p = 0.108). Including only first eyes in patients with bilateral cataract surgery yields similar results. There was no difference in the risk for pCME between learning surgeries and experienced surgeries focusing on eyes with PEX, patient’s age and surgeon’s sex. After excluding the first 100 surgeries for every surgeon in training similar results were observed (S1 Table).

**Table 2:** Results for multivariable logistic regression (generalized estimating equations model).

	pCME	No pCME	OR	95% CI		p-value
**OR Surgeon in training vs Experienced surgeon**	32 (17.4%)	2251 (8.9%)	1.58	1.03	2.10	0.034
	152 (82.6%)	22987 (91.1%)	Reference			
**OR F vs M**	103 (56.0%)	15436 (61.2%)	0.77	0.56	1.06	0.108
	81 (44.0%)	9802 (38.8%)	Reference			
**OR LE vs RE**	78 (42.4%)	12491 (49.5%)	0.74	0.57	0.96	0.022
	106 (57.6%)	12743 (50.5%)	Reference			
**OR PEX**	14 (7.6%)	1939 (7.7%)	0.82	0.42	1.62	0.572
	170 (92.4%)	23298 (92.3%)	Reference			
**OR Age**	76.5 (50.0, 91.0)	76.0 (50.0, 100.0)	1.00	0.99	1.02	0.784
**OR Duration of surgery**	12.6 (5.1, 30.0)	11.1 (3.0, 30.0)	1.04	1.02	1.07	0.002

Abbreviations: CI: confidence interval, F: female, LE: left eye, M: male, OR: odds ratio, pCME: pseudophakic cystoid macular edema, PEX: pseudo exfoliation syndrome, RE: right eye, vs: versus.

**Fig 1:**
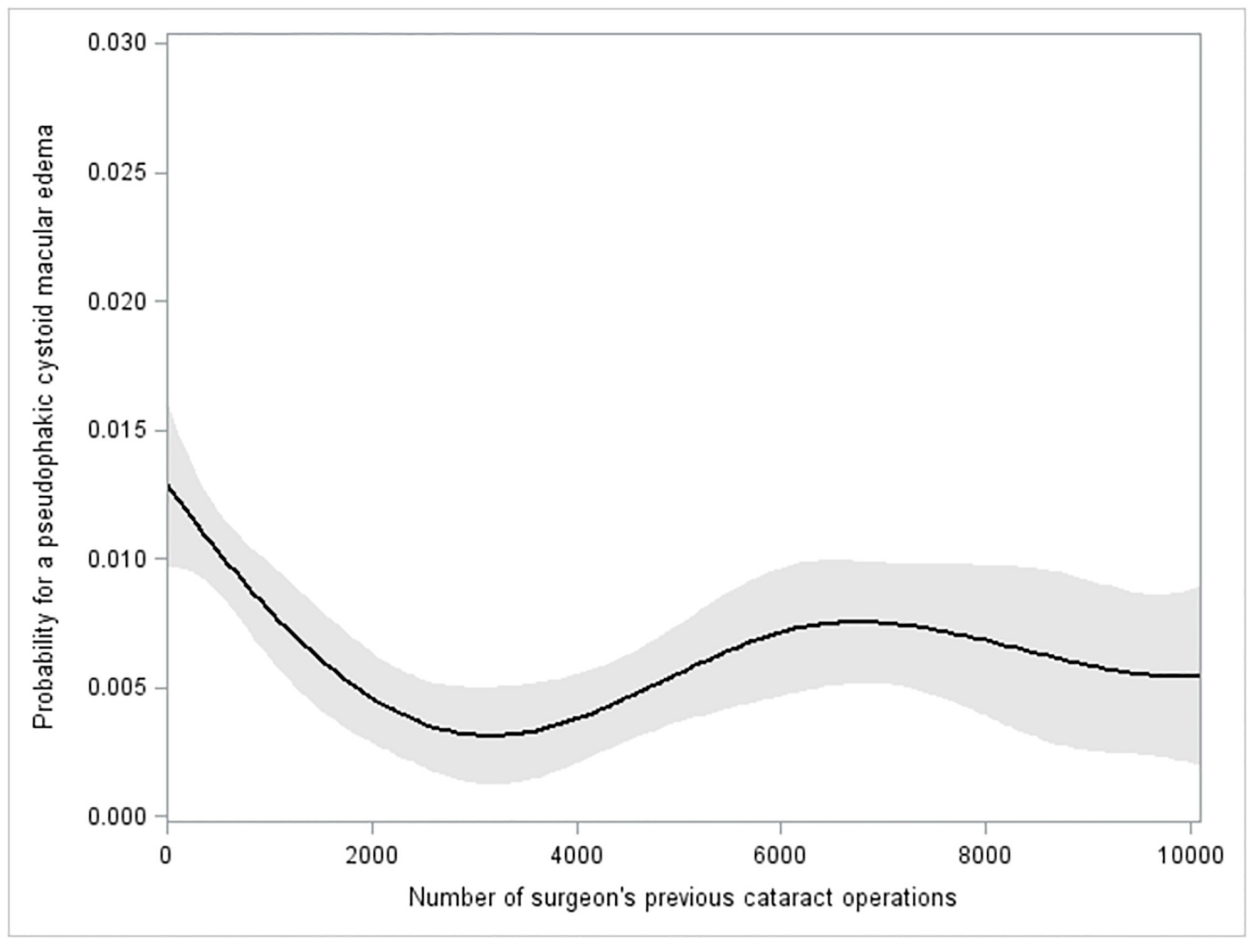
B-spline fit with three knots; shaded areas, 95% confidence limits. Between 0 to 10.000 surgeries.

Fig 1 shows the probability for a pCME on the y-axis and the running number of cataract surgeries/number of surgeon’s previous cataract surgeries on the x-axis. A B-spline fit shows first a decreasing probability for a pCME with increasing surgeon’s experience within the first 2500 procedures and then an increase after about 4000 performed surgeries.

## Discussion

In the present study, we analyzed the rate of pCME in association with surgical experience in phacoemulsification and lens implantation.

A learning curve in cataract surgery focusing on posterior capsule ruptures was already reported [24]. This study found a steadily falling rate of posterior capsular ruptures over the first 1500 procedures. In accordance, we found a steady decrease in the rate of pCME according to a surgeon’s expertise, but within the first 2500 procedures.

We observed a significant, 1.58 times higher chance for pCME after uneventful cataract surgery performed by surgeons in training compared to experienced surgeons (95% CI 1.03–2.10, p = 0.034).

Surgeries by surgeons in training were longer (median difference of about 6 minutes) and prolonged duration of surgery was associated with an increased risk for pCME (OR 1.06, 95% CI 1.03–1.09, p<0.001). Recently, Liebmann et al. showed that within the first 39 cases operative time decreased significantly with every additional case by -0.17 minutes, followed by a decrease between the 40 to 149 surgeries by -0.05 minutes with every additional surgery. No further significant improvement in surgery time was observed beyond the 150^th^ cataract surgery [34]. Phacoemulsification time and energy are known to be significantly correlated to visual impairment and development of pCME [35]. Although we could not evaluate effective phacoemulsifications times (EPT), longer duration of surgery possibly correlates to higher EPT and increased manipulation during surgery, being a risk factor for pCME itself.

In our study, surgeons in training more frequently performed cataract surgery on female patients (62.1%) with balanced side distribution (left eyes in 52.0%). However, the overall odds for pCME were lower for female patients (OR 0.77, p = 0.108) and surgeries on left eyes (OR 0.72, p = 0.022).

In Fig 1 we presented a B-spline fit. As illustrated, the rate for pCME steadily declined within the first 2500 surgeries before it flattens at around 3000 surgeries and then slightly increases again at 4000 surgeries until a plateau is reached after 6000 procedures. This configuration of the learning might be attributable to the fact that with increasing experience the probability for pCME decreases. The reason for the increase in the pCME rate among very experienced surgeons might have been caused by the fact that more difficult surgeries are commonly performed by more experienced surgeons. This is in accordance with Böhringer et al. who observed a steadily falling learning curve in the rate of posterior capsular ruptures over the first 1500 procedures, before it leveled off [24].

In our study, no association between PEX and pCME was observed. This is in accordance with observations from Shingleton et al. who found no difference of pCME in bilateral cataract surgery in patients with unilateral PEX [36].

We also analyzed the association between pCME and the surgeon’s sex by additionally including surgeon’s sex in the multivariable model. No association was found (female vs male: OR 1.03, 95% CI 0.72–1.49; p = 0.879). However, ratio of female surgeons is steadily increasing. The sex aspect in medical care is an issue of interest and has further gained importance in recent years. In recent studies by Gupta et al. and Gill et al., intraoperative complications in cataract surgery between female and male surgeons in training did not differ significantly [37, 38]. Hence, the number of women in surgical subspecialities in ophthalmology and the number of procedures completed by women shows disparities as reported by Gill et al. [39] And this is only one gender inequality, besides women in leadership, academics and research, pay gap, harassment, career satisfaction and mentorship. In Canada the number of surgeries performed by male surgeons compared to their female colleagues grew from 1.4 times to 1.7 times between 2000 to 2013. The age at entry is increasing and the percentage of early-career ophthalmologists performing cataract surgery declined [40]. Consenting, further studies reported on the lower cataract surgery volume by women, even after accounting for clinical volume, surgical experience and parental leave [38, 41–43].

We therefore encourage the statement by Sharoky et al. that a patient’s surgeons should be selected by experience rather than sex [44].

So far, learning curves in cataract surgery have been discussed frequently. According to previous results, phacoemulsification and capsulorhexis are the highest demanding steps in cataract surgery for residents [20, 45]. The number of intra- and postoperative complications was described to be steadily decreasing with increasing number of previous procedures [34, 46–48]. However, Al-Jindan et al. reported that after 40 procedures, proficiency levels were fairly satisfying [20]. Vedana et al. reported that it took 38 cataract surgeries until competency was achieved, assessing posterior capsule ruptures and best corrected visual acuity [21]. In contrast, Taravella et al. proposed an experience of 75 procedures until surgery is performed in a reasonable time without intervention or complication [22]. In accordance, Randleman et al. reported a significant reduction in vitreous loss and phacoemulsification time after the first 80 procedures [23]. However, we do not consent on the conclusion that 40 to 80 previous procedures are enough for a proficient cataract surgery. These numbers should rather be seen as a recommendation before supervision can be eased. As the results by Böhringer et al. and our results could show, learning curves are still ascending and complications rates still declining even after the number of 1000 previous procedures is exceeded [24].

During the study period between January 2010 and September 2018, the standardized postoperative therapeutic regimen consisted of glucocorticoids and antibiotics. However, no non-steroidal anti-inflammatory drugs (NSAIDs) were routinely given before October 2018 at our department. As it could be shown that the use of NSAIDs can prevent the occurrence of pCME after cataract surgery in nondiabetic patients [49], this might have influenced the occurrence of pCME in our study cohort. However, the postoperative therapeutic regimen was the same for both groups and should therefore impact both groups likewise. All patients with diabetes mellitus with and without any kind of diabetic retinopathy, known to have an increased risk for pCME, were excluded from this study.

This study has some limitations. We could only include patients with pCME who were either referred to our department or presented themselves with deteriorated vision or metamorphopsies. However, it concerns surgeries by surgeons in training and experienced surgeons similarly, we therefore assume that this does not influence our results. The Department of Ophthalmology, Medical University of Graz, where this study was conducted is a tertiary care center with a large catchment area. It is therefore assumable that patients with pCME are referred to our department. There are no data on visual acuity pre- and postoperatively. However, the aim of this study was not to compare visual outcomes between groups but to compare rates of postoperative pCME between surgeons in training and experienced surgeons.

In conclusion, the chance of pCME is significantly higher for surgeons in training but steadily decreasing within the first 2500 procedures, also associated to surgical time. No influence on the rate for pCME was found by comparing surgeon’s sex.

## Supporting information

S1 Table Results for multivariable logistic regression (generalized estimating equations model), excluding the first 100 procedures for surgeons in training. Abbreviations: CI: confidence interval, F: female, LE: left eye, M: male, OR: odds ratio, pCME: pseudophakic cystoid macular edema, PEX: pseudo exfoliation syndrome, RE: right eye, vs: versus.
(DOCX)
