# Supplementary material for: The impact of surgeon’s experience and sex on the incidence of cystoid macular edema after uneventful cataract surgery
Source: PLoS One. 2022 Dec 27;17(12):e0279518. doi: 10.1371/journal.pone.0279518 (PMC9794095; doi:10.1371/journal.pone.0279518)
Supplement: S1 Table — Abbreviations: CI: confidence interval, F: female, LE: left eye, M: male, OR: odds ratio, pCME: pseudophakic cystoid macular edema, PEX: pseudo exfoliation syndrome, RE: right eye, vs: versus. (DOCX) [file pone.0279518.s001.docx]

**Supporting Table 1: Results for multivariable logistic regression (generalized estimating equations model), excluding the first 100 procedures for surgeons in training**

|  | **pCME** | **No pCME** | **OR** | **95% CI** | | **p-value** |
| --- | --- | --- | --- | --- | --- | --- |
|  |  |  |  |  |  |  |
| **OR Surgeons in training vs Experienced Surgeon** | 28 (16.3%) | 2241 (9.1%) | 1.73 | 1.15 | 2.59 | 0.008 |
|  | 144 (83.7%) | 22268 (90.9%) | Reference | | |  |
| **OR F vs M** | 96 (55.8%) | 14976 (61.1%) | 0.78 | 0.56 | 1.08 | 0.132 |
|  | 76 (44.2%) | 9533 (38.9%) | Reference | | |  |
| **OR LE vs RE** | 73 (42.4%) | 12110 (49.4%) | 0.74 | 0.57 | 0.96 | 0.026 |
|  | 99 (57.6%) | 12395 (50.6%) | Reference | | |  |
| **OR PEX** | 13 (7.6%) | 1907 (7.8%) | 0.78 | 0.38 | 1.59 | 0.487 |
|  | 159 (92.4%) | 22601 (92.2%) | Reference | | |  |
| **OR Age** | 75.0 (50.0, 91.0) | 76.0 (50.0, 100.0) | 1.00 | 0.98 | 1.02 | 0.790 |
| **OR Duration of surgery** | 13.3 (5.1, 26.6) | 12.1 (3.0, 30.0) | 1.04 | 1.01 | 1.07 | 0.013 |

Abbreviations: CI: confidence interval, F: female, LE: left eye, M: male, OR: odds ratio, pCME: pseudophakic cystoid macular edema, PEX: pseudo exfoliation syndrome, RE: right eye, vs: versus.
