# Supplementary material for: The impact of surgeon’s experience and sex on the incidence of cystoid macular edema after uneventful cataract surgery
Source: PLoS One. 2022 Dec 27;17(12):e0279518. doi: 10.1371/journal.pone.0279518 (PMC9794095; doi:10.1371/journal.pone.0279518)
Supplement: Attachment — Submitted filename: Response to the Reviewers.docx [file pone.0279518.s002.docx]

Dear Prof. Grzybowski, MD, PhD, MBA!

thank you very much for the thorough review of our manuscript and giving us the opportunity to make corrections according to the valuable recommendations of the reviewers. Three reviewers have carefully examined our paper, raising points that help us to improve our manuscript as it is currently present.

PLOS ONE would be a most optimal venue to communicate our findings to the global audience in clinical science.

The corrections we made in the paper are listed below. The corrections are highlighted in the marked-up copy version of the manuscript.

We also implemented the journal requirements. The manuscript now meets PLOS ONE’s style requirements. Ethical approval is given for the study and stated in the manuscript. The Ethics Committee of the Medical University of Graz ruled that written informed consent was not required for this study from each participant. All relevant data are presented in the manuscript. Patient-level data contain potentially sensitive information and are not publicly available in a repository due to restrictions based upon privacy and ethical regulations.

***************************************************************************

**Reviewer #1**

**Reviewer #1 writes:** “This interesting manuscript demonstrated that the surgeon's little experience increases the chance of cystoid macular edema in cataract surgery with intraocular lens implantation. The segmented analysis of surgeons with more than 100 surgeries further reinforced this thesis”

**Author response:**

We thank the reviewer for this comment on our manuscript.

**Reviewer #2 writes:** “This paper is quite ambitious in its goal because of the large number of parameters included to evaluate the risk of pCME after cataract surgery performed by trainees versus experienced surgeons. Most readers will start with a guess that indeed they expect to have higher pCME in trainees compared to experienced surgeons. This is also the conclusion of the authors.
However, this paper would still have been of interest provided the study parameters studied were precisely defined.
The idea is to study pCME (psuedophakic CME), however, the chapter on materials and methods does not define when the OCT of the macula was performed and whether this was performed systematically. It becomes clear for the reader when reading the discussion (page 17 line320-322) that pCME has not been studied as such but clinically significant CME has been studied. Patients were referred when presenting visual complaints and when the visual disturbances could be objectivated by means of OCT images.
This is a major point that needs to be clearly stated form the start.”

**Author response:**

We thank the reviewer for reviewing our manuscript and the very important comments to improve this study! With this response, we hope to implement your recommendations and your experience into our manuscript.
Due to the retrospective nature of the study and the standardized postoperative management we were not able to perform OCT scans on every patient at predefined follow-ups. After discharge from the hospital on the day of surgery patients are referred to a postoperative examination at a registered ophthalmologist within two to five days. However, patients with persisting cystoid macular edema or visual deterioration are usually referred to our acute day ward, where OCT scans of the macula and if required fluorescein- and indocyanine green angiographies are performed. Although not all postoperative CME will be screened with this approach, we assume that the loss of diagnosed patients will affect both study groups in the same manner.
Spectral domain OCTs (Heidelberg Engineering, Heidelberg, Germany were conducted using volume scanning with 25 sections covering a field of 20x20° in the macular region. A built-in eye tracking software ensured exact position of the recorded scans. Sections were received using the high-speed mode with a resolution of 7 μm axially x 14μm laterally and a distance of 240 μm between sections. CME was defined as macular thickness >300 μm and the presence of intraretinal hyporeflective cysts within the ETDRS (Early Treatment Diabetic Retinopathy Study) circle.
This information was provided in the methods section of the manuscript.

**Reviewer #2 writes:** “Page 4 line 73: the numbers 2.61 to 5.05 are ODDS ratios?”

**Author response:**

The numbers 2.61 to 5.05 declare an increase of the relative risk. This missing information was added to the corresponding lines.

**Reviewer #2 writes:** “Page 6 line 104: surgical time is not defined. starting point-end point? Phako time? Please specify”

**Author response:**

The surgical time is defined as the time difference from the beginning of the surgery (first incision of the cornea) until the end of the surgery (after intracameral injection of cefuroxime). This was added to the corresponding line in the manuscript.

**Reviewer #2 writes:** “Page 7 line 109: We do understand it is a retrospective study, needing approval of the Ethical committee but without the need for signed patient's informed consent. I suppose this is what the authors wanted to explain in their paper?”

**Author response:**

Thank you very much for this correction. We certainly obtained approval from the local ethical committee, but waiver was given for the necessity for written informed consent.

**Reviewer #2 writes:** “Page 7 line 127: There is no clear-cut definition of which surgeon is considered trainee or experienced surgeon. A trainee is per definition an MD who is in training to become ophthalmologist. However, the reader gets the impression that the authors consider a trainee somebody who is already ophthalmologist but in training to become senior cataract surgeon. I think the authors mix the terms of trainee, fellow and sub-specialist in cataract and refractive surgeon. It would have been much clearer if the authors would have considered clear differences in the two surgeon groups studied. This remains a very week point.”

**Author response:**

According to a previous study by Böhringer et al. we defined a cut-off for “unexperienced” surgeons named as surgeons in training or trainees and “experienced” surgeons at 300 surgeries. The German Ophthalmological Society recommended that the first 300 cataract surgeries should be done under supervision before enough experience is acquired. We analyzed this threshold for the occurrence of pCME. The terms “trainee” and “experienced surgeon” therefore do not represent the level of medical training but surgical experience. Surgeries by “trainees” in our manuscript corresponds to a “training surgery” by Böhringer et al. This obscurity was clarified in the manuscript. The term “trainee” was changed to “surgeon in training” in the manuscript.

**Reviewer #2 writes:** “Page 9 line 172-174: Is PEX an indication for trainees?”

**Author response:**

The presence of pseudo exfoliation syndrome was documented for every surgery in both groups and analyzed as a risk factor for pCME. Trainees and experienced surgeons were doing the surgeries on patients with PEX.

**Reviewer #2 writes:** “Page 11 line 206: babies are also included in the case series (age ranging from 0- 100) of experienced surgeons. babies, children and young adults should have been excluded from this study”

**Author response:**

Thank you very much for this remark. It is of course not intended to include babies, children, and young adults but only patients with senile cataracts. We therefore changed the inclusion criteria to patient’s age ranging from 50 years to 100 years.

**Reviewer #2 writes:** “Page 15 line 277-289: the surgeon's sex discussion from the literature is inappropriately related to ophthalmology.”

**Author response:**

The discussion on gender in cataract surgery volume and complication rates was changed. Recent and relevant literature was included and the discussion on complications in specialties other than ophthalmology was removed.

**Reviewer #3 writes:** “The authors retrospectively compared rate of CME after cataract surgery between surgeons of different sex and experience. The drawback is related to retrospective nature of the study. As you have not performed OCT, you can not say how many patients have CME. You can just say that from those patients that had related complaints, 188 patients had CME.”

**Author response:**

Thank you for this remark. Duo to the retrospective design of the study and aim of the study we did not perform standardized OCT of the macula for every patient. We therefore only report on clinically significant edema of patients who were referred to our clinics. Hence, the number of patients with pCME does not describe an incidence. We changed the sentence “In total, pCME occurred in 188 eyes …” to “In total, pCME was reported in 188 eyes …”.

**Reviewer #3 writes:** “The second draw back is entering both eyes of some patients and one eye of some patients that make inter-eye correlation study necessary that you have not performed.”

**Author response:**

To account for inter-eye correlation in the data, Generalized Estimating Equation (GEE) models with exchangeable working correlation structure were used. This approach is suggested by Ying et al (PMID: 28532207). However, including only one eye per patient (the first documented eye) yields similar results (table below). The reference was changed in the materials and methods section and the information was added in the results section.

| **multivariable logistic regression** | **Odds Ratio** | **95% Confidence Interval** | | **p-value** |  |
| --- | --- | --- | --- | --- | --- |
|  |  |  |  |  |  |
| **OR Trainee vs Experienced surgeon** | 1.99 | 1.24 | 3.19 | 0.004 |  |
| **OR F vs M** | 0.73 | 0.52 | 1.04 | 0.086 |  |
| **OR LE vs RE** | 0.87 | 0.61 | 1.23 | 0.424 |  |
| **OR PEX vs no PEX** | 0.80 | 0.39 | 1.65 | 0.543 |  |
| **OR Age** | 1.00 | 0.98 | 1.02 | 0.883 |  |
| **OR Duration of surgery** | 1.04 | 1.00 | 1.07 | 0.034 |  |
