# Supplementary material for: The impact of surgeon’s experience and sex on the incidence of cystoid macular edema after uneventful cataract surgery
Source: PLoS One. 2022 Dec 27;17(12):e0279518. doi: 10.1371/journal.pone.0279518 (PMC9794095; doi:10.1371/journal.pone.0279518)
Supplement: Peer review history

# Peer review: Decision Letter 0

**DOI:** 10.1371/journal.pone.0279518.r001

**Authors:** Andrzej Grzybowski

19 Jul 2022

PONE-D-22-14684The Impact of Surgeon’s Experience and Sex on the Incidence of Cystoid Macular Edema after uneventful Cataract SurgeryPLOS ONE

Dear Dr. List,

Thank you for submitting your manuscript to PLOS ONE. After careful consideration, we feel that it has merit but does not fully meet PLOS ONE’s publication criteria as it currently stands. Therefore, we invite you to submit a revised version of the manuscript that addresses the points raised during the review process.

Please submit your revised manuscript by Sep 02 2022 11:59PM. If you will need more time than this to complete your revisions, please reply to this message or contact the journal office at plosone@plos.org. When you're ready to submit your revision, log on to https://www.editorialmanager.com/pone/ and select the 'Submissions Needing Revision' folder to locate your manuscript file.

Please include the following items when submitting your revised manuscript:

- A rebuttal letter that responds to each point raised by the academic editor and reviewer(s). You should upload this letter as a separate file labeled 'Response to Reviewers'.
- A marked-up copy of your manuscript that highlights changes made to the original version. You should upload this as a separate file labeled 'Revised Manuscript with Track Changes'.
- An unmarked version of your revised paper without tracked changes. You should upload this as a separate file labeled 'Manuscript'.

If you would like to make changes to your financial disclosure, please include your updated statement in your cover letter. Guidelines for resubmitting your figure files are available below the reviewer comments at the end of this letter.

If applicable, we recommend that you deposit your laboratory protocols in protocols.io to enhance the reproducibility of your results. Protocols.io assigns your protocol its own identifier (DOI) so that it can be cited independently in the future. For instructions see: https://journals.plos.org/plosone/s/submission-guidelines#loc-laboratory-protocols. Additionally, PLOS ONE offers an option for publishing peer-reviewed Lab Protocol articles, which describe protocols hosted on protocols.io. Read more information on sharing protocols at https://plos.org/protocols?utm_medium=editorial-email&utm_source=authorletters&utm_campaign=protocols.

We look forward to receiving your revised manuscript.

Kind regards,

Andrzej Grzybowski

Academic Editor

PLOS ONE

Journal Requirements:

When submitting your revision, we need you to address these additional requirements.

1. Please ensure that your manuscript meets PLOS ONE's style requirements, including those for file naming. The PLOS ONE style templates can be found at 

https://journals.plos.org/plosone/s/file?id=wjVg/PLOSOne_formatting_sample_main_body.pdf and 

https://journals.plos.org/plosone/s/file?id=ba62/PLOSOne_formatting_sample_title_authors_affiliations.pdf

2. You indicated that ethical approval was not necessary for your study. We understand that the framework for ethical oversight requirements for studies of this type may differ depending on the setting and we would appreciate some further clarification regarding your research. Could you please provide further details on why your study is exempt from the need for approval and confirmation from your institutional review board or research ethics committee (e.g., in the form of a letter or email correspondence) that ethics review was not necessary for this study? Please include a copy of the correspondence as an "Other" file.

3. We note that you have indicated that data from this study are available upon request. PLOS only allows data to be available upon request if there are legal or ethical restrictions on sharing data publicly. For more information on unacceptable data access restrictions, please see http://journals.plos.org/plosone/s/data-availability#loc-unacceptable-data-access-restrictions. 

In your revised cover letter, please address the following prompts:

a) If there are ethical or legal restrictions on sharing a de-identified data set, please explain them in detail (e.g., data contain potentially sensitive information, data are owned by a third-party organization, etc.) and who has imposed them (e.g., an ethics committee). Please also provide contact information for a data access committee, ethics committee, or other institutional body to which data requests may be sent.

b) If there are no restrictions, please upload the minimal anonymized data set necessary to replicate your study findings as either Supporting Information files or to a stable, public repository and provide us with the relevant URLs, DOIs, or accession numbers. For a list of acceptable repositories, please see http://journals.plos.org/plosone/s/data-availability#loc-recommended-repositories.

We will update your Data Availability statement on your behalf to reflect the information you provide.

[Note: HTML markup is below. Please do not edit.]

Reviewers' comments:

Reviewer's Responses to Questions

**Comments to the Author**

1. Is the manuscript technically sound, and do the data support the conclusions?

The manuscript must describe a technically sound piece of scientific research with data that supports the conclusions. Experiments must have been conducted rigorously, with appropriate controls, replication, and sample sizes. The conclusions must be drawn appropriately based on the data presented. 

Reviewer #1: Yes

Reviewer #2: No

Reviewer #3: Yes

**********

2. Has the statistical analysis been performed appropriately and rigorously? 

Reviewer #1: Yes

Reviewer #2: Yes

Reviewer #3: Yes

**********

3. Have the authors made all data underlying the findings in their manuscript fully available?

The PLOS Data policy requires authors to make all data underlying the findings described in their manuscript fully available without restriction, with rare exception (please refer to the Data Availability Statement in the manuscript PDF file). The data should be provided as part of the manuscript or its supporting information, or deposited to a public repository. For example, in addition to summary statistics, the data points behind means, medians and variance measures should be available. If there are restrictions on publicly sharing data—e.g. participant privacy or use of data from a third party—those must be specified.

Reviewer #1: Yes

Reviewer #2: No

Reviewer #3: Yes

**********

4. Is the manuscript presented in an intelligible fashion and written in standard English?

PLOS ONE does not copyedit accepted manuscripts, so the language in submitted articles must be clear, correct, and unambiguous. Any typographical or grammatical errors should be corrected at revision, so please note any specific errors here.

Reviewer #1: Yes

Reviewer #2: Yes

Reviewer #3: Yes

**********

5. Review Comments to the Author

Please use the space provided to explain your answers to the questions above. You may also include additional comments for the author, including concerns about dual publication, research ethics, or publication ethics. (Please upload your review as an attachment if it exceeds 20,000 characters)

Reviewer #1: this interesting manuscript demonstrated that the surgeon's little experience increases the chance of cystoid macular edema in cataract surgery with intraocular lens implantation.

The segmented analysis of surgeons with more than 100 surgeries further reinforced this thesis

Reviewer #2: This paper is quite ambitious in its goal because of the large number of parameters included to evaluate the risk of pCME after cataract surgery performed by trainees versus experienced surgeons. Most readers will start with a guess that indeed they indeed expect to have higher pCME in trainees compared to experienced surgeons. This is also the conclusion of the authors.

However, this paper would still have been of interest provided the study parameters studied were precisely defined.

The idea is to study pCME (psuedophakic CME), however, the chapter on materials and methods does not define when the OCT of the macula was performed and whether this was performed systematically. It becomes clear for the reader when reading the discussion (page 17 line320-322) that pCME has not been studied as such but clinically significant CME has been studied. Patients were referred when presenting visual complaints and when the visual disturbances could be objectivated by means of OCT images.

This is a major point that needs to be clearly stated form the start.

Page 4 line 73: the numbers 2.61 to 5.05 are ODDS ratios?

Page 6 line 104: surgical time is not defined. starting point-end point? Phako time? Please specify

Page 7 line 109: We do understand it is a retrospective study, needing approval of the Ethical committee but without the need for signed patient's informed consent. I suppose this is what the authors wanted to explain in their paper?

Page 7 line 127: There is no clear-cut definition of which surgeon is considered trainee or experienced surgeon. A trainee is per definition an MD who is in training to become ophthalmologist. However, the reader gets the impression that the authors consider a trainee somebody who is already ophthalmologist but in training to become senior cataract surgeon. I think the authors mix the terms of trainee, fellow and sub-specialist in cataract and refractive surgeon. It would have been much clearer if the authors would have considered clear differences in the two surgeon groups studied. This remains a very week point.

Page 9 line 172-174: Is PEX an indication for trainees?

Page 11 line 206: babies are also included in the case series (age ranging from 0- 100) of experienced surgeons. babies, children and young adults should have been excluded from this study

Page 15 line 277-289: the surgeon's sex discussion from the literature is inappropriately related to ophthalmology.

Reviewer #3: The authors retrospectively compared rate of CME after cataract surgery between surgeons of different sex and experience. The drawback is related to retrospective nature of the study. As you have not performed OCT, you can not say how many patients have CME. You can just say that from those patients that had related complaints, 188 patients had CME.

The second draw back is entering both eyes of some patients and one eye of some patients that make inter-eye correlation study necessary that you have not performed.

**********

6. PLOS authors have the option to publish the peer review history of their article (what does this mean?). If published, this will include your full peer review and any attached files.

If you choose “no”, your identity will remain anonymous but your review may still be made public.

**Do you want your identity to be public for this peer review?** For information about this choice, including consent withdrawal, please see our Privacy Policy.

Reviewer #1: **Yes: **RODRIGO PESSOA CAVALCANTI LIRA

Reviewer #2: No

Reviewer #3: **Yes: **Hesam Hashemian

**********

[NOTE: If reviewer comments were submitted as an attachment file, they will be attached to this email and accessible via the submission site. Please log into your account, locate the manuscript record, and check for the action link "View Attachments". If this link does not appear, there are no attachment files.]

While revising your submission, please upload your figure files to the Preflight Analysis and Conversion Engine (PACE) digital diagnostic tool, https://pacev2.apexcovantage.com/. PACE helps ensure that figures meet PLOS requirements. To use PACE, you must first register as a user. Registration is free. Then, login and navigate to the UPLOAD tab, where you will find detailed instructions on how to use the tool. If you encounter any issues or have any questions when using PACE, please email PLOS at figures@plos.org. Please note that Supporting Information files do not need this step.

# Author comment: Author response to Decision Letter 0

**DOI:** 10.1371/journal.pone.0279518.r002

23 Oct 2022

Dear Prof. Grzybowski, MD, PhD, MBA!

thank you very much for the thorough review of our manuscript and giving us the opportunity to make corrections according to the valuable recommendations of the reviewers. Three reviewers have carefully examined our paper, raising points that help us to improve our manuscript as it is currently present.

PLOS ONE would be a most optimal venue to communicate our findings to the global audience in clinical science.

The corrections we made in the paper are listed below. The corrections are highlighted in the marked-up copy version of the manuscript.

We also implemented the journal requirements. The manuscript now meets PLOS ONE’s style requirements. Ethical approval is given for the study and stated in the manuscript. The Ethics Committee of the Medical University of Graz ruled that written informed consent was not required for this study from each participant. All relevant data are presented in the manuscript. Patient-level data contain potentially sensitive information and are not publicly available in a repository due to restrictions based upon privacy and ethical regulations.

***************************************************************************

Reviewer #1

Reviewer #1 writes: “This interesting manuscript demonstrated that the surgeon's little experience increases the chance of cystoid macular edema in cataract surgery with intraocular lens implantation. The segmented analysis of surgeons with more than 100 surgeries further reinforced this thesis”

Author response:

We thank the reviewer for this comment on our manuscript.

Reviewer #2 writes: “This paper is quite ambitious in its goal because of the large number of parameters included to evaluate the risk of pCME after cataract surgery performed by trainees versus experienced surgeons. Most readers will start with a guess that indeed they expect to have higher pCME in trainees compared to experienced surgeons. This is also the conclusion of the authors.

However, this paper would still have been of interest provided the study parameters studied were precisely defined.

The idea is to study pCME (psuedophakic CME), however, the chapter on materials and methods does not define when the OCT of the macula was performed and whether this was performed systematically. It becomes clear for the reader when reading the discussion (page 17 line320-322) that pCME has not been studied as such but clinically significant CME has been studied. Patients were referred when presenting visual complaints and when the visual disturbances could be objectivated by means of OCT images.

This is a major point that needs to be clearly stated form the start.”

Author response:

We thank the reviewer for reviewing our manuscript and the very important comments to improve this study! With this response, we hope to implement your recommendations and your experience into our manuscript.

Due to the retrospective nature of the study and the standardized postoperative management we were not able to perform OCT scans on every patient at predefined follow-ups. After discharge from the hospital on the day of surgery patients are referred to a postoperative examination at a registered ophthalmologist within two to five days. However, patients with persisting cystoid macular edema or visual deterioration are usually referred to our acute day ward, where OCT scans of the macula and if required fluorescein- and indocyanine green angiographies are performed. Although not all postoperative CME will be screened with this approach, we assume that the loss of diagnosed patients will affect both study groups in the same manner.

Spectral domain OCTs (Heidelberg Engineering, Heidelberg, Germany were conducted using volume scanning with 25 sections covering a field of 20x20° in the macular region. A built-in eye tracking software ensured exact position of the recorded scans. Sections were received using the high-speed mode with a resolution of 7 μm axially x 14μm laterally and a distance of 240 μm between sections. CME was defined as macular thickness >300 μm and the presence of intraretinal hyporeflective cysts within the ETDRS (Early Treatment Diabetic Retinopathy Study) circle.

This information was provided in the methods section of the manuscript.

Reviewer #2 writes: “Page 4 line 73: the numbers 2.61 to 5.05 are ODDS ratios?”

Author response:

The numbers 2.61 to 5.05 declare an increase of the relative risk. This missing information was added to the corresponding lines.

Reviewer #2 writes: “Page 6 line 104: surgical time is not defined. starting point-end point? Phako time? Please specify”

Author response:

The surgical time is defined as the time difference from the beginning of the surgery (first incision of the cornea) until the end of the surgery (after intracameral injection of cefuroxime). This was added to the corresponding line in the manuscript.

Reviewer #2 writes: “Page 7 line 109: We do understand it is a retrospective study, needing approval of the Ethical committee but without the need for signed patient's informed consent. I suppose this is what the authors wanted to explain in their paper?”

Author response:

Thank you very much for this correction. We certainly obtained approval from the local ethical committee, but waiver was given for the necessity for written informed consent.

Reviewer #2 writes: “Page 7 line 127: There is no clear-cut definition of which surgeon is considered trainee or experienced surgeon. A trainee is per definition an MD who is in training to become ophthalmologist. However, the reader gets the impression that the authors consider a trainee somebody who is already ophthalmologist but in training to become senior cataract surgeon. I think the authors mix the terms of trainee, fellow and sub-specialist in cataract and refractive surgeon. It would have been much clearer if the authors would have considered clear differences in the two surgeon groups studied. This remains a very week point.”

Author response:

According to a previous study by Böhringer et al. we defined a cut-off for “unexperienced” surgeons named as surgeons in training or trainees and “experienced” surgeons at 300 surgeries. The German Ophthalmological Society recommended that the first 300 cataract surgeries should be done under supervision before enough experience is acquired. We analyzed this threshold for the occurrence of pCME. The terms “trainee” and “experienced surgeon” therefore do not represent the level of medical training but surgical experience. Surgeries by “trainees” in our manuscript corresponds to a “training surgery” by Böhringer et al. This obscurity was clarified in the manuscript. The term “trainee” was changed to “surgeon in training” in the manuscript.

Reviewer #2 writes: “Page 9 line 172-174: Is PEX an indication for trainees?”

Author response:

The presence of pseudo exfoliation syndrome was documented for every surgery in both groups and analyzed as a risk factor for pCME. Trainees and experienced surgeons were doing the surgeries on patients with PEX.

Reviewer #2 writes: “Page 11 line 206: babies are also included in the case series (age ranging from 0- 100) of experienced surgeons. babies, children and young adults should have been excluded from this study”

Author response:

Thank you very much for this remark. It is of course not intended to include babies, children, and young adults but only patients with senile cataracts. We therefore changed the inclusion criteria to patient’s age ranging from 50 years to 100 years.

Reviewer #2 writes: “Page 15 line 277-289: the surgeon's sex discussion from the literature is inappropriately related to ophthalmology.”

Author response:

The discussion on gender in cataract surgery volume and complication rates was changed. Recent and relevant literature was included and the discussion on complications in specialties other than ophthalmology was removed.

Reviewer #3 writes: “The authors retrospectively compared rate of CME after cataract surgery between surgeons of different sex and experience. The drawback is related to retrospective nature of the study. As you have not performed OCT, you can not say how many patients have CME. You can just say that from those patients that had related complaints, 188 patients had CME.”

Author response:

Thank you for this remark. Duo to the retrospective design of the study and aim of the study we did not perform standardized OCT of the macula for every patient. We therefore only report on clinically significant edema of patients who were referred to our clinics. Hence, the number of patients with pCME does not describe an incidence. We changed the sentence “In total, pCME occurred in 188 eyes …” to “In total, pCME was reported in 188 eyes …”.

Reviewer #3 writes: “The second draw back is entering both eyes of some patients and one eye of some patients that make inter-eye correlation study necessary that you have not performed.”

Author response:

To account for inter-eye correlation in the data, Generalized Estimating Equation (GEE) models with exchangeable working correlation structure were used. This approach is suggested by Ying et al (PMID: 28532207). However, including only one eye per patient (the first documented eye) yields similar results (table below). The reference was changed in the materials and methods section and the information was added in the results section.

Attachment Submitted filename: Response to the Reviewers.docx Click here for additional data file.

# Peer review: Decision Letter 1

**DOI:** 10.1371/journal.pone.0279518.r003

**Authors:** Andrzej Grzybowski

8 Dec 2022

The Impact of Surgeon’s Experience and Sex on the Incidence of Cystoid Macular Edema after uneventful Cataract Surgery

PONE-D-22-14684R1

Dear Dr. List,

We’re pleased to inform you that your manuscript has been judged scientifically suitable for publication and will be formally accepted for publication once it meets all outstanding technical requirements.

Within one week, you’ll receive an e-mail detailing the required amendments. When these have been addressed, you’ll receive a formal acceptance letter and your manuscript will be scheduled for publication.

An invoice for payment will follow shortly after the formal acceptance. To ensure an efficient process, please log into Editorial Manager at http://www.editorialmanager.com/pone/, click the 'Update My Information' link at the top of the page, and double check that your user information is up-to-date. If you have any billing related questions, please contact our Author Billing department directly at authorbilling@plos.org.

If your institution or institutions have a press office, please notify them about your upcoming paper to help maximize its impact. If they’ll be preparing press materials, please inform our press team as soon as possible -- no later than 48 hours after receiving the formal acceptance. Your manuscript will remain under strict press embargo until 2 pm Eastern Time on the date of publication. For more information, please contact onepress@plos.org.

Kind regards,

Andrzej Grzybowski

Academic Editor

PLOS ONE

Additional Editor Comments (optional):

Thank you for preparing a good revision.

Reviewers' comments: 

Reviewer's Responses to Questions

**Comments to the Author**

1. If the authors have adequately addressed your comments raised in a previous round of review and you feel that this manuscript is now acceptable for publication, you may indicate that here to bypass the “Comments to the Author” section, enter your conflict of interest statement in the “Confidential to Editor” section, and submit your "Accept" recommendation.

Reviewer #3: All comments have been addressed

**********

2. Is the manuscript technically sound, and do the data support the conclusions?

The manuscript must describe a technically sound piece of scientific research with data that supports the conclusions. Experiments must have been conducted rigorously, with appropriate controls, replication, and sample sizes. The conclusions must be drawn appropriately based on the data presented. 

Reviewer #3: Yes

**********

3. Has the statistical analysis been performed appropriately and rigorously? 

Reviewer #3: Yes

**********

4. Have the authors made all data underlying the findings in their manuscript fully available?

The PLOS Data policy requires authors to make all data underlying the findings described in their manuscript fully available without restriction, with rare exception (please refer to the Data Availability Statement in the manuscript PDF file). The data should be provided as part of the manuscript or its supporting information, or deposited to a public repository. For example, in addition to summary statistics, the data points behind means, medians and variance measures should be available. If there are restrictions on publicly sharing data—e.g. participant privacy or use of data from a third party—those must be specified.

Reviewer #3: Yes

**********

5. Is the manuscript presented in an intelligible fashion and written in standard English?

PLOS ONE does not copyedit accepted manuscripts, so the language in submitted articles must be clear, correct, and unambiguous. Any typographical or grammatical errors should be corrected at revision, so please note any specific errors here.

Reviewer #3: Yes

**********

6. Review Comments to the Author

Please use the space provided to explain your answers to the questions above. You may also include additional comments for the author, including concerns about dual publication, research ethics, or publication ethics. (Please upload your review as an attachment if it exceeds 20,000 characters)

Reviewer #3: The main drawback as mentioned in the first review is the weak retrospective design and failure to document cases of CME with OCT.

**********

7. PLOS authors have the option to publish the peer review history of their article (what does this mean?). If published, this will include your full peer review and any attached files.

If you choose “no”, your identity will remain anonymous but your review may still be made public.

**Do you want your identity to be public for this peer review?** For information about this choice, including consent withdrawal, please see our Privacy Policy.

Reviewer #3: No

**********

# Editor report: Acceptance letter

**DOI:** 10.1371/journal.pone.0279518.r004

**Authors:** Andrzej Grzybowski

15 Dec 2022

PONE-D-22-14684R1 

The impact of surgeon’s experience and sex on the incidence of cystoid macular edema after uneventful cataract surgery 

Dear Dr. List:

I'm pleased to inform you that your manuscript has been deemed suitable for publication in PLOS ONE. Congratulations! Your manuscript is now with our production department. 

If your institution or institutions have a press office, please let them know about your upcoming paper now to help maximize its impact. If they'll be preparing press materials, please inform our press team within the next 48 hours. Your manuscript will remain under strict press embargo until 2 pm Eastern Time on the date of publication. For more information please contact onepress@plos.org.

If we can help with anything else, please email us at plosone@plos.org. 

Thank you for submitting your work to PLOS ONE and supporting open access. 

Kind regards, 

PLOS ONE Editorial Office Staff

on behalf of

Dr. Andrzej Grzybowski 

Academic Editor

PLOS ONE
